# Celastrol, an NF-κB Inhibitor, Improves Insulin Resistance and Attenuates Renal Injury in db/db Mice

**DOI:** 10.1371/journal.pone.0062068

**Published:** 2013-04-26

**Authors:** Jung Eun Kim, Mi Hwa Lee, Deok Hwa Nam, Hye Kyoung Song, Young Sun Kang, Ji Eun Lee, Hyun Wook Kim, Jin Joo Cha, Young Youl Hyun, Sang Youb Han, Kum Hyun Han, Jee Young Han, Dae Ryong Cha

**Affiliations:** 1 Department of Internal Medicine, Division of Nephrology, Korea University, Ansan City, Kyungki-Do, Korea; 2 Department of Internal Medicine, Division of Nephrology, Wonkwang University, Gunpo City, Kyungki-Do, Korea; 3 Department of Internal Medicine, Division of Nephrology, Sungkyunkwan University, Seoul, Korea; 4 Department of Internal Medicine, Division of Nephrology, Inje University, Goyang City, Kyungki-Do, Korea; 5 Department of Pathology, Inha University, Incheon City, Kyungki-Do, Korea; College of Tropical Agriculture and Human Resources, University of Hawaii, United States of America

## Abstract

The NF-κB pathway plays an important role in chronic inflammatory and autoimmune diseases. Recently, NF-κB has also been suggested as an important mechanism linking obesity, inflammation, and metabolic disorders. However, there is no current evidence regarding the mechanism of action of NF-κB inhibition in insulin resistance and diabetic nephropathy in type 2 diabetic animal models. We investigated the effects of the NF-κB inhibitor celastrol in db/db mice. The treatment with celastrol for 2 months significantly lowered fasting plasma glucose (FPG), HbA1C and homeostasis model assessment index (HOMA-IR) levels. Celastrol also exhibited significant decreases in body weight, kidney/body weight and adiposity. Celastrol reduced insulin resistance and lipid abnormalities and led to higher plasma adiponectin levels. Celastrol treatment also significantly mitigated lipid accumulation and oxidative stress in organs including the kidney, liver and adipose tissue. The treated group also exhibited significantly lower creatinine levels and urinary albumin excretion was markedly reduced. Celastrol treatment significantly lowered mesangial expansion and suppressed type IV collagen, PAI-1 and TGFβ1 expressions in renal tissues. Celastrol also improved abnormal lipid metabolism, oxidative stress and proinflammatory cytokine activity in the kidney. In cultured podocytes, celastrol treatment abolished saturated fatty acid-induced proinflammatory cytokine synthesis. Taken together, celastrol treatment not only improved insulin resistance, glycemic control and oxidative stress, but also improved renal functional and structural changes through both metabolic and anti-inflammatory effects in the kidney. These results suggest that targeted therapy for NF-κB may be a useful new therapeutic approach for the management of type II diabetes and diabetic nephropathy.

## Introduction

Type 2 diabetes mellitus is the leading cause of end-stage renal disease and is associated with morbidity and mortality due to cardiovascular disease. The increased mortality in type 2 diabetes mellitus is partially due to insulin resistance [1]. From a clinical perspective, insulin resistance is frequently combined with hyperinsulinemia, abnormal glucose metabolism, hypertension, atherosclerosis and dyslipidemia; collectively these conditions are referred to as metabolic syndrome [2], [3].

Although the pathogenic mechanism of diabetic nephropathy is complex, inflammatory mechanisms may play important roles in the initiation and progression of diabetic nephropathy [4], [5]. Macrophage infiltration, activation of inflammatory cytokines and adhesion molecules in the diabetic kidney have been reported in both human and animal diabetic models in a manner similar to other immunologic renal diseases [6], [7], [8].

NF-kappaB (NF-κB) is a ubiquitous and well-known transcription factor responsible for regulating the expressions of genes that are involved in inflammatory pathways such as proinflammatory cytokines, chemokines and adhesion molecules [9], [10]. Since NF-κB plays a pivotal role in the inflammatory process, NF-κB has been an important and attractive therapeutic target for the management of many inflammatory diseases. Increasing evidence demonstrates that NF-κB is activated and contributes to macrophage infiltration in experimental models of diabetic kidney disease [11], [12], [13]. In addition, recent studies suggest that high glucose, mechanical stretching, angiotensin II and proteinuria contribute to NF-κB activation [13], [14], [15].

In terms of insulin resistance, NF-κB activation in adipose tissue has recently been implicated as an important mechanism in the development of insulin resistance [16]. Obesity is accompanied by the infiltration and activation of macrophages in adipose tissue, leading to chronic inflammation of adipose tissue [17]. Adipose tissue is an important organ in obesity-induced inflammation, since obesity induces phenotypic changes in adipocytes such as hypertrophy, and also induces an inflammatory response in adipocytes in an autocrine or paracrine fashion, resulting in impaired adipocyte function [18]. However, the roles of this pathway in diabetic nephropathy and insulin resistance have not been clearly delineated.

In this study we investigated the effect of celastrol, an NF-κB inhibitor, on insulin resistance and diabetic nephropathy under the hypothesis that inhibition of the NF-κB pathway may improve insulin resistance and renal function through the modulation of inflammatory processes in both adipose tissues and kidneys in db/db mice.

## Materials and Methods

### Animal experiments

Six-week-old male non-diabetic *db/m* and diabetic *db/db* mice (C57BLKS/J-*lepr* ^db^/*lepr* ^db^) were purchased from the Jackson Laboratory (Sacramento, CA, USA). The mice were given free access to food and tap water and were caged individually under controlled temperature (23±2°C) and humidity (55±5%) with an artificial light cycle. At 8 weeks of age, mice were divided into 3 groups. Group 1 consisted of non-diabetic control *db/m* mice (n = 8), group 2 consisted of *db/db* mice as a control group (n = 8), and group 3 consisted of *db/db* mice that were treated via injection with 1 mg/kg/day of celastrol (Sigma. St. Louis, MO, USA) intraperitoneally for 2 months (n = 8). In addition, we performed another in vivo experiment to evaluate whether celastrol could potentially have significant effect on food intake and body weight in non-diabetic control *db/m* mice. Control non-diabetic *db/m* mice were divided into two groups with or without treatment with 1 mg/kg/day of celastrol for 2 weeks (n = 5 in each group), and compare the physical parameters after 2weeks. During experiments, food intake, water intake, urine volume, body weight, fasting plasma glucose concentration, and HbA1c levels were measured monthly. Plasma glucose levels were measured by a glucose oxidase-based method and creatinine levels were determined using an HPLC method. Plasma insulin levels and plasma adiponectin levels were measured using an ELISA kit (Linco Research, St. Charles, MO, USA). Plasma triglyceride and cholesterol analyses were measured using a GPO-Trinder kit (Sigma, St. Louis, MO, USA). Plasma lipoprotein profiles were measured using a fast protein liquid chromatography (HPLC) system. The blood levels of hemoglobin A1c (HbA1c) were calculated by an IN2IT system (Bio-Rad, UK). The homeostasis model assessment index (HOMA-IR) was calculated by the following equation: fasting glucose (mmol/L) × fasting insulin (mU/L)/22.5. An insulin-tolerance test (ITT) was performed to assess the insulin resistance state. ITT was performed following 8-hour fasting and blood samples were collected through the tail vein. Mice received 0.75 unit/kg of regular insulin by i.p. injection and blood sampling was done to measure blood glucose levels at 0, 30, 60, 90, and 120 min after insulin injection. To determine urinary microalbumin excretion, individual mice were placed in a metabolic cage and urine was collected for 24 h. The urinary microalbumin concentration was determined by a competitive enzyme-linked immunosorbent assay (Shibayagi, Shibukawa, Japan) and corrected by urinary creatinine concentration. Plasma and urinary levels of 8-isoprostane were measured using an ELISA kit (Cayman Chemical, Ann Arbor, MI, USA). Lipids from the hepatic, adipose, and renal cortical tissues were extracted as described by Bligh and Dyer [19]. Total cholesterol and triglyceride contents were measured using a commercial kit (Wako Chemicals, Richmond, VA, USA). The extent of peroxidative reaction in the hepatic, adipose, and kidney tissues was determined by directly measuring lipid hydroperoxides (LPOs) using redox reactions with ferrous ions from tissue homogenates and an LPO assay kit (Cayman Chemical, Ann Arbor, MI, USA), as described previously [20]. At the end of the study period, systolic blood pressure was measured using tail-cuff plethysmography (LE 5001-Pressure Meter, Letica SA, Barcelona, Spain). Mice were sacrificed under anesthesia by i.p. injection of sodium pentobarbital (50 mg/kg). This study was carried out in strict accordance with the recommendations in the Guide for the Care and Use of Laboratory Animals of the National Institutes of Health. The protocol was approved by the Committee on the Ethics of Animal Experiments of the University of Korea (Permit Number: KUIACUC-20111001-1).

### Analysis of gene expression by real-time quantitative PCR

Total RNA was extracted from experimental cells with Trizol reagent and further purified using an RNeasy Mini kit (Qiagen, Valencia, CA, USA). Primers were designed from the respective gene sequences using Primer 3 software and the secondary structures of templates were examined and excluded using the mfold software program. Table S1 shows the nucleotide sequences of the primers. Quantitative gene expression was performed on a Light Cycler 1.5 system (Roche Diagnostics Corporation, Indianapolis, IN, USA) using SYBR Green technology. Real-time reverse transcription-PCR was performed for 10 min at 50°C and 5 min at 95°C. Subsequently, 30–35 cycles were applied, consisting of denaturation for 10 s at 95°C and annealing with extension for 30 s at 60°C. At the end of the PCR cycle, samples were heated to 95°C to check that a single PCR product was obtained. The ratio of each gene and β-actin level (relative gene expression number) was calculated by subtracting the threshold cycle number (Ct) of the target gene from that of β -actin and raising 2 to the power of this difference.

### Histopathological evaluation and immunohistochemistry

Cardiac, hepatic, and adipose tissues were fixed for 48 h with 10% paraformaldehyde at 4°C, dehydrated, embedded in paraffin, cut into 4 µm thick slices, and stained with periodic acid-Schiff (PAS), Masson's trichrome (MT), and hematoxylin and eosin. Glomerular mesangial expansion was scored semiquantitatively and the percentage of mesangial matrix occupying each glomerulus was rated from 0 to 4 as follows: 0, 0%; 1, <25%; 2, 25–50%; 3, 50–75%; and 4, >75%. For immunohistochemical staining, renal tissues were sliced into 4 µm sections. Slides were transferred to a 10 mmol/L citrate buffer solution at a pH of 6.0. Various staining conditions were then applied as follows: sections were microwaved for 10–20 min to retrieve antigens for TGF-β1 staining, transferred to Biogenex Retrievit (pH 8.0) (InnoGenex, San Ramon, CA, USA) for PAI-1 staining or treated with trypsin (Sigma, St Louis, MO, USA) for 30 min at 37°C for type IV collagen staining. After washing in water, 3.0% H_2_O_2_ in methanol was applied for 10 min in order to block endogenous peroxidase activity and the slides were incubated at room temperature for 40 min with normal goat serum (TGF-β1and type IV collagen) or 10% power block (PAI-1) to prevent nonspecific detection. Next, slides were incubated at 4°C overnight with a primary antibody including rabbit polyclonal anti-type IV collagen antibody (1∶200; Santa Cruz Biotechnology, Santa Cruz, CA, USA), a rabbit polyclonal anti-TGF-β1 antibody (1∶200; Santa Cruz Biotechnology) and a rabbit anti-PAI-1 antibody (1∶50; American Diagnostica, Stanford, CT, USA). Slides were incubated in a secondary antibody for 30 min. For coloration, slides were incubated at room temperature with a mixture of 0.05% 3,3-diaminobenzidine containing 0.01% H_2_O_2_ and counterstained with Mayer's hematoxylin. Negative control sections were stained under identical conditions with the buffer solution substituting for the primary antibody. In order to evaluate the results of immunohistochemical staining, glomerular fields were graded semiquantitatively under a high-power field containing 50–60 glomeruli and an average score was calculated as described previously [21].

### Western blot analysis

Nuclear and cytoplasmic proteins were extracted from renal cortical tissues and cells using a commercial nuclear extraction kit according to the manufacturer's instructions (Active Motif, Carlsbad, CA). Under denaturing conditions, 35 µg of protein were electrophoresed on a 10% SDS-PAGE mini-gel. Proteins were transferred onto a polyvinylidene difluoride membrane (Immobilon-P; Millipore, Bedford, MA, USA) for 60 min at 100 V. After incubation in blocking solution (Tris-buffered saline containing 150 mM NaCl, 50 nM Tris, 0.05% Tween-20 and 5% BSA, pH 7.5) for 1 h at room temperature, the membrane was hybridized in blocking buffer with goat polyclonal anti-TLR4 antibody (1∶500, Santa Cruz Biotechnology, CA, USA), anti-NOX4 antibody (1∶500, Novus Biologicals, USA), rabbit monoclonal anti- NF-κB p65 antibody(1∶2000, Cell signaling, USA), rabbit monoclonal anti-α-tubulin antibody (1∶1000,Cell Signaling Technology) or mouse monoclonal anti-β-actin antibody (1∶ 10000, Sigma Aldrich) overnight at 4°C with gentle shaking. Afterward, peroxidase conjugated secondary antibodies (1∶5000) were applied for 1 h at room temperature, followed by reaction with chemiluminescence (ECL) reagent (Amersham, Buckinghamshire, UK).

### Fatty acid analysis in hepatic and adipose tissues

Fatty acid compositions in hepatic and adipose tissue were analyzed at the end of study period by gas chromatography-flame ionization detection (GC-FID) method on a HP6890N GC-FID with a hydrogen flame ionization detector and an Supelco^TM^ SP-2560 column (100 m×0.25 mm×0.20 µm). Helium served as the carrier gas, and 1 µl sample was loaded when the injection temperature was 260°C. Briefly, an extraction with chloroform was conducted. The dry extracts were dissolved in a few drops of chloroform and filled in thin liquid chromatography plates for separation of the lipids. The lipid esters were trans-methylated and the methyl esters were extracted. The FA methyl esters were separated by gas–liquid chromatography (GLC). The FAs were identified by comparison of the retention times of separation, controlled by SUPELCO^TM^37 component FAME Mix (47885-U). Thirty seven fatty acids including saturated (SFA), monounsaturated (MUFA), and polyunsaturated (PUFA) fatty acids were identified and quantified.

### Podocyte culture experiment

Since podocytes are the major target cells affected by high glucose and free fatty acid stimulation in the diabetic milieu, we used podocytes to further define the molecular mechanism of NF-κB inhibition on proinflammatory cytokine synthesis. A thermosensitive, SV 40-transfected immortalized mouse podocyte cell line that had been obtained as a generous gift from Peter Mundel (Albert Einstein college of medicine, N.Y.) was used for this study [22]. All cells were grown in a type I collagen coated dish (Iwaki, Tokyo, Japan) supplemented with RPMI media containing heat inactivated 10% fetal calf serum (Invitrogen, Carlsbad, CA), penicillin, and streptomycin. Differentiated podocytes were grown to subconfluence in the growth media and then cultured for 24 hours in a medium containing 5 mmol/L D-glucose and 1% FCS before being exposed to experimental conditions. The normal glucose group (NG group) used confluent cell monolayers cultured with 5 mmol/L of D-glucose, while the high glucose group (HG group) used 30 mmol/L of D-glucose. To evaluate the different effects of saturated (SFA), and monounsaturated (MUFA) fatty acids on proinflammatory cytokine synthesis, we treated some wells with palmitic acid (SFA) at a final concentration of 100 µM (Sigma, St. Louis, MO, USA) or oleic acid (PUFA) at a final concentration of 10 µM (Sigma, St. Louis, MO, USA) under either normal or high glucose conditions. To elucidate the effect of NF-κB inhibition, 1 µM of celastrol was added to the cells for 1 h before treatment with free fatty acid. All experimental groups were cultured in triplicate and harvested at 12 hours for extraction of the total RNA and secretory cytokine proteins. The secreted proinflammatory cytokines were measured in the culture supernatants using Millipore's MILLIPLEX™ Mouse Cytokine/Chemokine kit and the supernatant levels of cytokines were expressed relative to the total protein concentration. All experiments were performed in triplicate and cells were harvested at 12 hours to extract total RNA and protein.

### Statistical analysis

A nonparametric analysis was used because of the relatively small number of samples. Results were expressed as mean ± standard error of the mean (SEM). Multiple comparisons were performed using the Kruskal-Wallis test with Bonferroni correction, followed by a Mann-Whitney U-test using a microcomputer-assisted program with SPSS for Windows 10.0 (SPSS, Chicago, IL, USA). P<0.05 was considered statistically significant.

## Results

### Effects of celastrol on biochemical and physical parameters in experimental animals

Table 1 shows biochemical results for each group. As expected, body weight, food intake, water intake, plasma insulin levels and fasting plasma glucose concentrations were significantly higher in diabetic *db/db* mice than those of non-diabetic *db/m* mice throughout the study period. Additionally, urine volume, organ mass of kidney, fat and liver was significantly higher in diabetic *db/db* mice compared with those of non-diabetic *db/m* mice. However, there were no significant differences in plasma levels of creatinine, adiponectin and 8-isoprostane, and systolic blood pressure between non-diabetic *db/m* mice and diabetic *db/db* mice. Interestingly, body weight was significantly lower in the celastrol-treated group due to decreases in food intake. In terms of organ mass change, celastrol treatment did not induce any significant changes in the weights of the liver or heart. Epididymal fat mass and kidney weight were significantly lower in the celastrol treatment group compared with the control group. However, there were no significant differences in systolic blood pressure or plasma levels of insulin or isoprostane between the two groups. Celastrol treatment induced a marked improvement in glycemic control. Even after only 4 weeks of treatment, fasting plasma glucose levels were significantly decreased; this hypoglycemia was persistently observed until the end of the study (Table 1). Furthermore, plasma adiponectin levels were significantly higher in the celastrol-treated group. In addition, we also performed another in vivo experiment in *db/m* mice to define celastrol also decrease food intake and body weight independent of diabetic status. As shown in Table S2, 2weeks treatment with celastrol also induced a significant decrease in food intake and body weight in *db/m* mice.

**Table 1:** Physical and biochemical parameters of experimental animals.

Parameters	week	*db/m* control	*db/db* + vehicle	*db/db* + celastrol
Body weight (g)	0	24.28±0.18	33.2±0.41^***^	33.4±0.81^***^
	4	29.14±0.40	45.7±1.29^***^	38.0±1.26^***,‡^
	8	29.00±0.53	46.5±1.94^***^	37.2±2.22^***,†^
Daily Food intake (g)	0	3.53±0.31	4.68±0.26	4.95±0.53
	4	3.14±0.09	4.93±0.08^***^	3.85±0.24^**,‡^
	8	2.97±0.17	5.06±0.18^***^	4.10±0.46^**,†^
Daily water intake (g)	0	5.35±0.25	7.87±0.22^***^	7.45±0.69^**^
	4	3.98±0.22	16.45±0.47^***^	4.45±0.43^***,‡^
	8	3.60±0.16	17.56±0.54^***^	8.14±0.62^**,‡^
Fasting plasma glucose (mmol/l)	0	9.3±0.5	18.2±1.9^**^	19.4±3.4^**^
	4	8.3±0.3	35.7±1.8^***^	17.2±4.2^*,‡^
	8	8.59±0.32	32.1±1.8^***^	16.2±3.1^*,‡^
UV (ml/day)	0	0.45±0.08	0.66±0.10	0.71±0.11
	4	0.39±0.08	0.75±0.12^*^	0.68±0.03^*^
	8	0.39±0.05	1.42±0.32^**^	0.76±0.14
Kidney/100 g BW	8	0.49±0.02	1.36±0.05^***^	0.52±0.03^‡^
Heart/100 g BW	8	0.36±0.02	0.38±0.02	0.35±0.02
Fat/100 g BW	8	1.15±0.08	6.08±0.41^***^	2.73±0.74^*,‡^
Liver/100 g BW	8	4.17±0.63	7.26±0.56^**^	7.58±1.13^**^
P-adiponectin ( µg/ml)	8	2.89±0.20	2.51±0.05	29.1±2.44^**,‡^
P- 8-isoprostane(pg/ml)	8	249±29.8	473±74.4	814±172
P-creatinine ( µmol/l)	8	35.0±4.0	45.0±6.0	29.0±2.0^†^
P-insulin (ng/ml)	8	1.71±0.38	9.73±0.81^***^	8.80±2.41^**^
SBP (mmHg)	8	118±16	115±15	124±11

UV, urine volume; P, plasma. Values are expressed as means ± SEM. Statistical analysis was performed between groups at the same time periods; ^*^P<0.05; ^**^P<0.01; ^***^P<0.001 vs. *db/m* control; ^†^P<0.05; ^‡^P<0.01 vs. *db/db* + vehicle.

### Effects of celastrol on metabolic parameters in experimental animals

**Figure 1:**
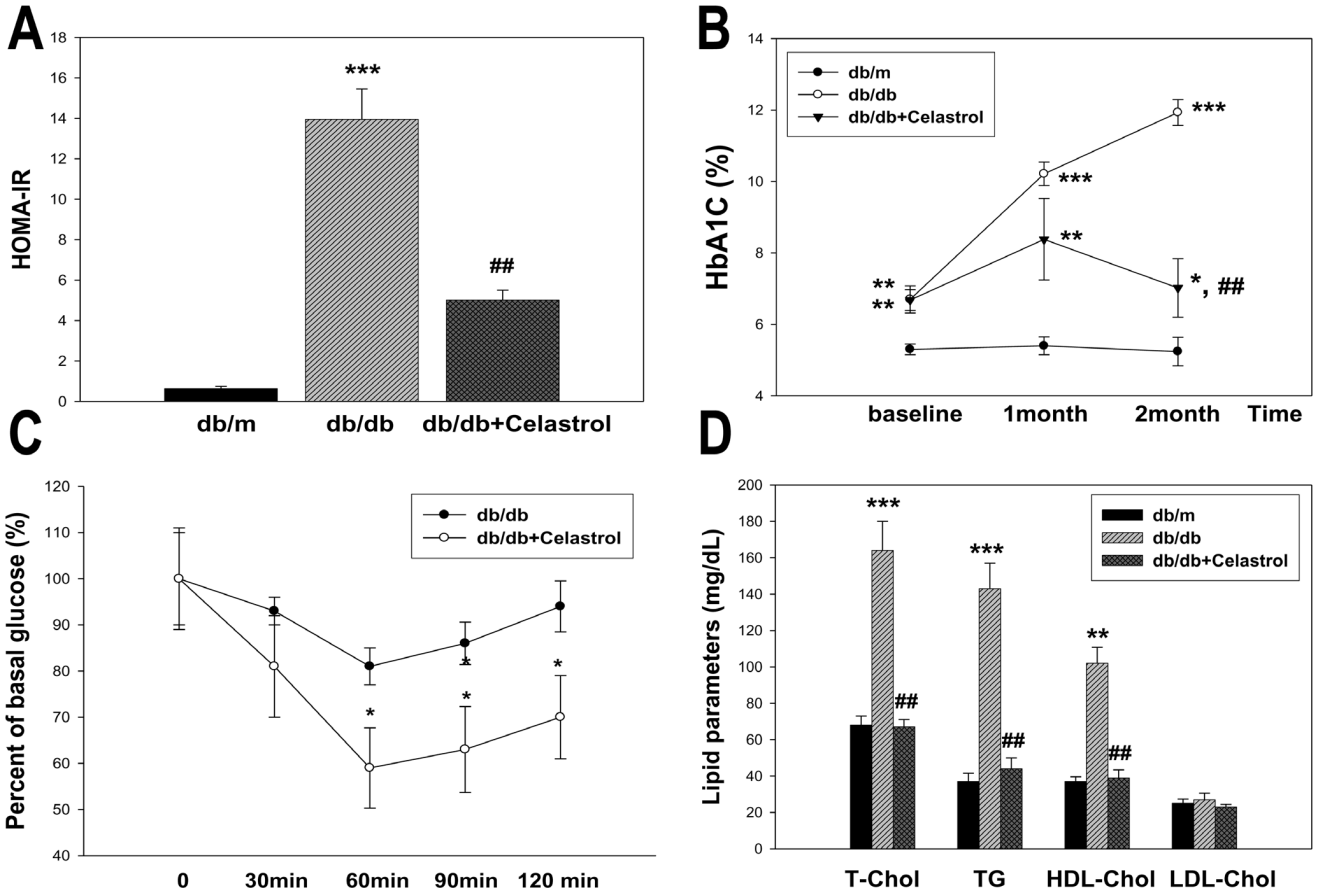
Effects of celastrol on HOMA-IR, HbA1C, insulin resistance and plasma lipid levels in experimental animals. Data are shown as the mean±SEM; *p<0.05, **p<0.01, ***p<0.001 vs. *db/m* group; ##p<0.01 vs. control *db/db* group.

Celastrol treatment induced a marked improvement in insulin resistance. As shown in Figures 1A and 1B, levels of HOMA-IR and HbA1C were significantly decreased after celastrol treatment. The improvement in insulin resistance by celastrol treatment was further confirmed by intraperitoneal ITT (Figure 1C). In accordance with the improved insulin resistance, celastrol treatment significantly decreased plasma total cholesterol and triglyceride levels (Figure 1D).

### Effects of celastrol on renal function and histological changes in experimental animals

**Figure 2:**
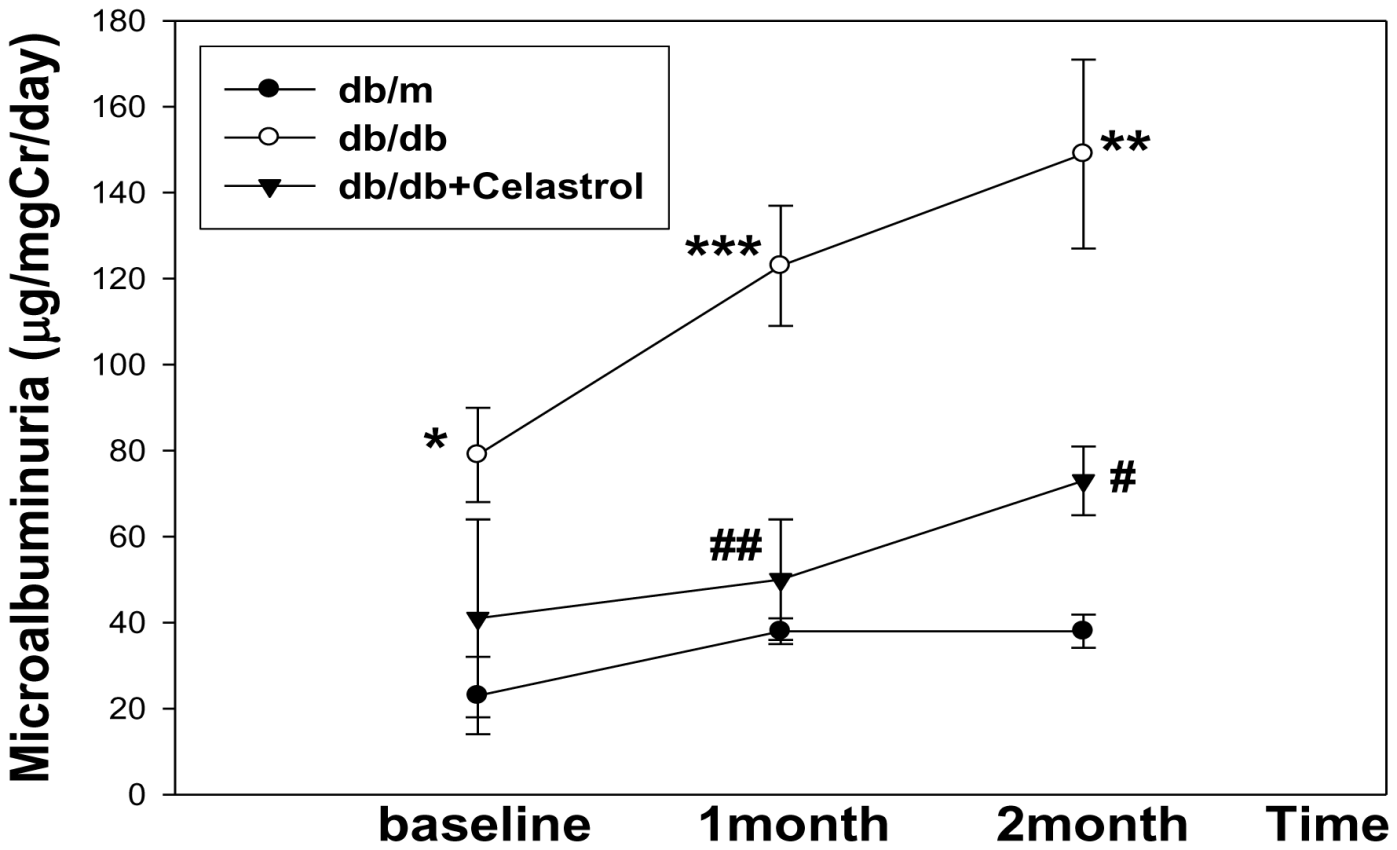
Effects of celastrol on urinary albumin excretion in experimental animals. Data are shown as the mean±SEM; *p<0.05, **p<0.01, ***p<0.001 vs. *db/m* group; #p<0.05; ##p<0.01 vs. control *db/db* group.

**Figure 3:**
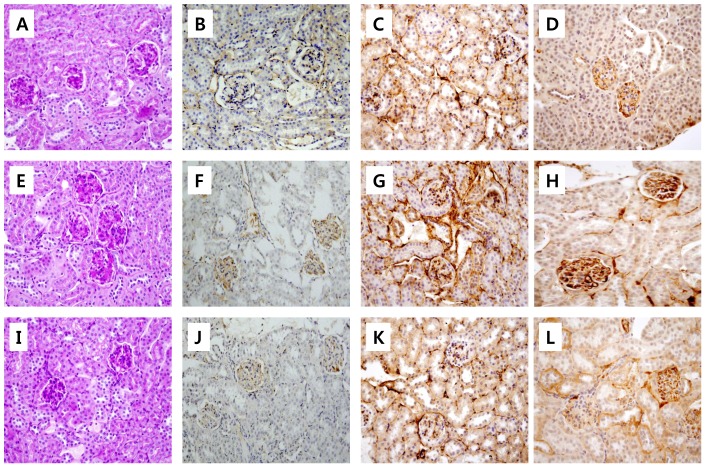
Representative renal histologic findings in experimental animals. (A, E, I) PAS, (B, F, J) TGFβ-1, (C, G, K) Type IV collagen, (D, H, L) PAI-1; A–D, *db/m*; E–H, *db/db*; I–L, *db/db*+celastrol. Original magnification X400.

**Figure 4:**
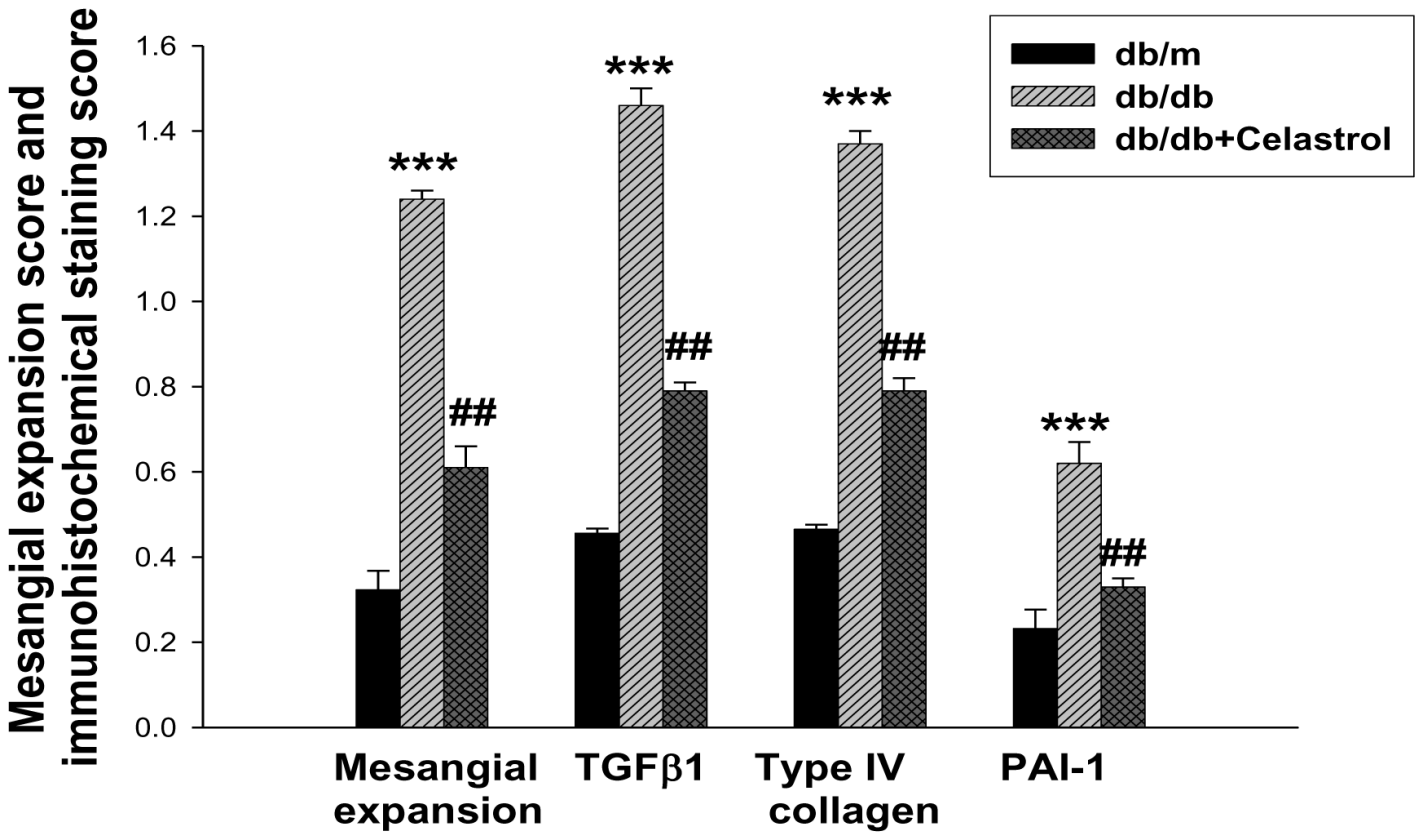
Glomerular mesangial expansion score and immunostaining score. Data are shown as the mean±SEM; ***p<0.001 vs. *db/m* group; ##p<0.01 vs. control *db/db* group.

Urinary albumin excretion was significantly higher in diabetic *db/db* mice than those of non-diabetic *db/m* mice throughout the study period (Figure 2). Celastrol treatment markedly decreased urinary albumin excretion after 4 weeks of treatment and this effect was persistently observed until the end of the study period (Figure 2). In terms of renal function, the celastrol group also showed significantly lower levels of plasma creatinine levels (Table 1). Consistent with marked attenuation of albuminuria, celastrol treatment significantly suppressed the expression of profibrotic and proinflammatory molecules in the kidney. Figures 3A–3L show representative renal pathology and immunostaining for TGFβ1, type IV collagen, and PAI-1. Consistent with marked attenuation of albuminuria, mesangial expansion was more severe in diabetic *db/db* mice than non-diabetic *db/m* mice, and mesangial expansion was markedly improved in the celastrol treatment group (Figure 3A, 3E, 3I, Figure 4). Furthermore, the immunostaining scores for TGFβ1, type IV collagen and PAI-1, major fibrotic molecules in fibrotic glomeruli, also demonstrated dramatic improvement in the celastrol-treated group (Figure 3, 4).

### Effects of celastrol on oxidative stress and inflammatory cytokines in experimental animals

**Figure 5:**
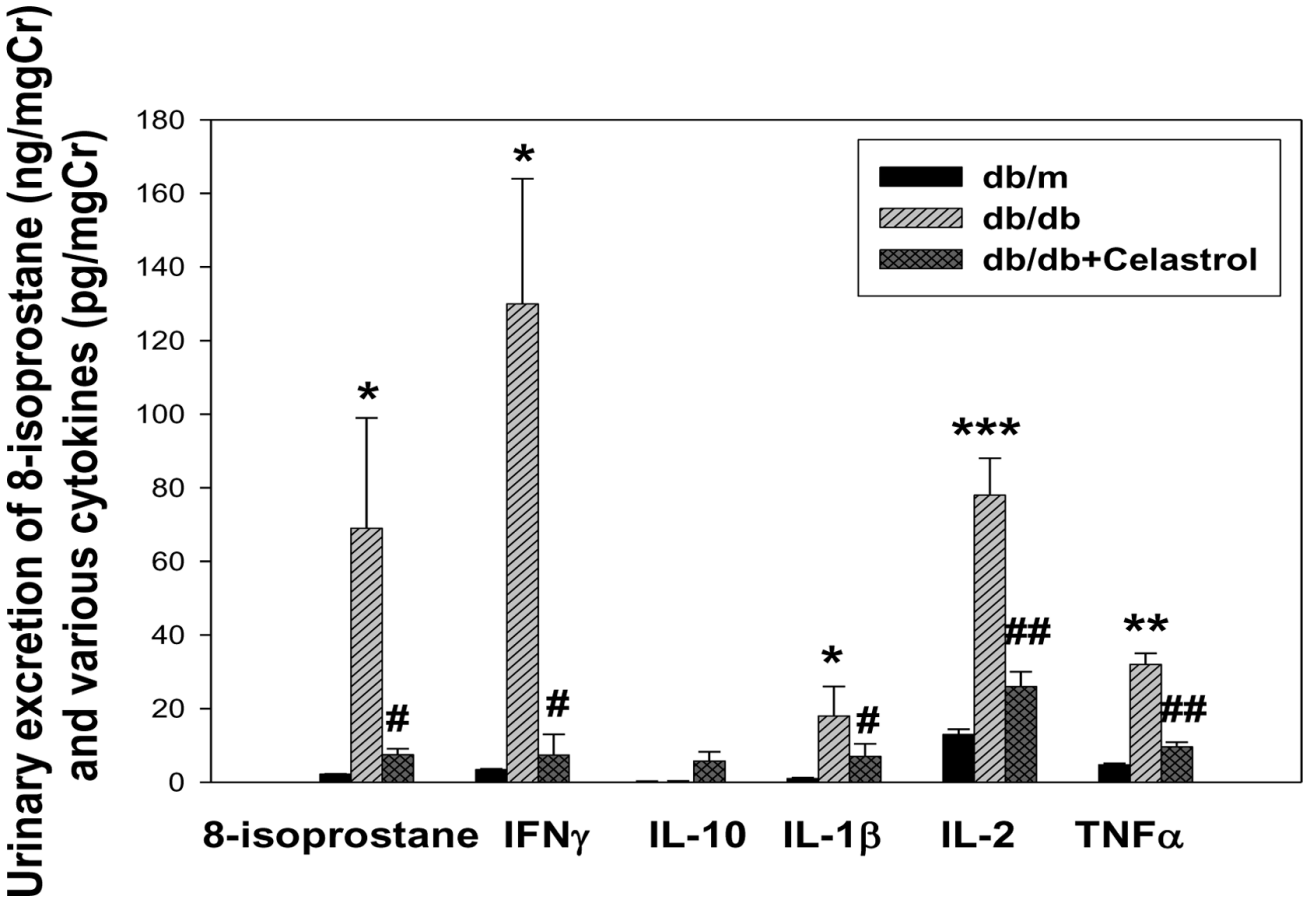
Effects of celastrol on urinary levels of cytokines in experimental animals. Data are shown as the mean±SEM; *p<0.05, **p<0.01, ***p<0.001 vs. *db/m* group; #p<0.05, ##p<0.01 vs. control *db/db* group.

**Figure 6:**
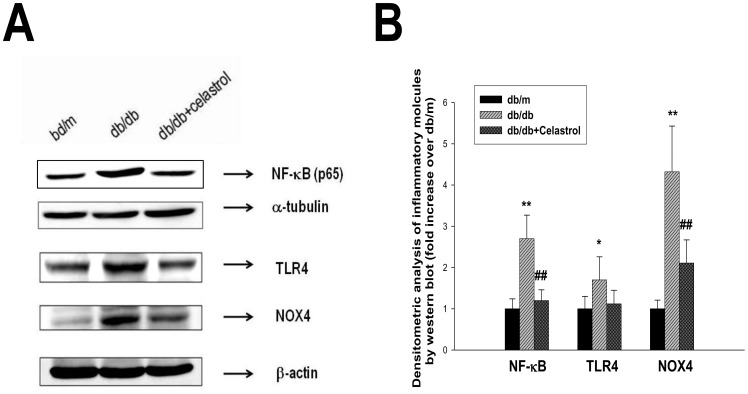
Effects of celastrol on inflammatory molecules in experimental animals. (A) Representative western blot for NF-κB p65, TLR4, and NOX4 protein in renal cortical tissues in experimental animals. (B) Densitometric analysis of western results. Data are shown as the mean±SEM; *p<0.05, **p<0.01, vs. *db/m* group; ##p<0.01 vs. control *db/db* group.

Since celastrol treatment improved albuminuria and structural changes in diabetic kidneys and NF-κB inhibition can inhibit proinflammatory cytokine production, we next examined whether improvement in renal function was derived from the suppression of proinflammatory cytokines in the kidney. As shown in Figure 5, urinary excretion of inflammatory cytokines was markedly higher in diabetic *db/db* mice than non-diabetic *db/m* mice, and celastrol treatment markedly suppressed the urinary levels of these cytokines. Additionally, urinary levels of 8-isoprostane, an indicator of oxidative stress in the kidney, also profoundly decreased in celastrol-treated group (Figure 5). In addition, we performed western blot using renal cortical tissues for the NF-κB to elucidate whether the NF-κB is overexpressed in diabetic kidneys. As shown in Figure 6, diabetic mice showed significantly higher levels of activation in NF-κB compared with control *db/m* mice as determined by the nuclear expressions of the p65 subunit of NF-κB, toll-like receptor 4 (TLR4) and NADPH oxidase 4 (NOX4) in renal cortical tissues. Celastrol treatment significantly suppressed inflammatory molecules in diabetic kidney (Figure 6A, 6B)

### Effects of celastrol on fatty acid composition in hepatic and adipose tissues in experimental animals

Table S3 shows the change in fatty acids composition in hepatic and adipose tissues after 2months treatment with celastrol. In non-diabetic control *db/m* mice, most FAs were composed of n-6 PUFAs (42%), SFAs (35%) and MUFAs (22%) in fat, and n-6 PUFAs (72%), n-3 PUFAs (16%) and SFAs (11%) in liver. However, FAs composition in diabetic *db/db* mice showed significantly higher levels of SFAs and lower levels of n-6 PUFAs in fat and liver compared with those in control *db/m* mice. Interestingly, celastrol treated *db/db* mice showed significantly lower levels of SFAs, and higher levels of MUFAs in fat and liver compared with those in diabetic *db/db* mice (Table S3).

### Effects of celastrol on histological changes in liver and adipose tissue

Figure S1 shows the representative adipose tissue and liver pathology in the experimental groups at the end of the study period. In accordance with improved plasma lipid abnormalities, celastrol treatment markedly decreased hepatic steatosis (Figure S1F). Interestingly, adipose tissue obtained from epididymal fat revealed that celastrol treatment induced phenotypic changes in adipocytes, causing differentiation into small adipocytes (Figure S1C). Further, this phenotypic change was consistent with the change in epididymal fat mass that was decreased by celastrol treatment.

### Effects of celastrol on oxidative stress and lipid accumulation in experimental animals

**Figure 7:**
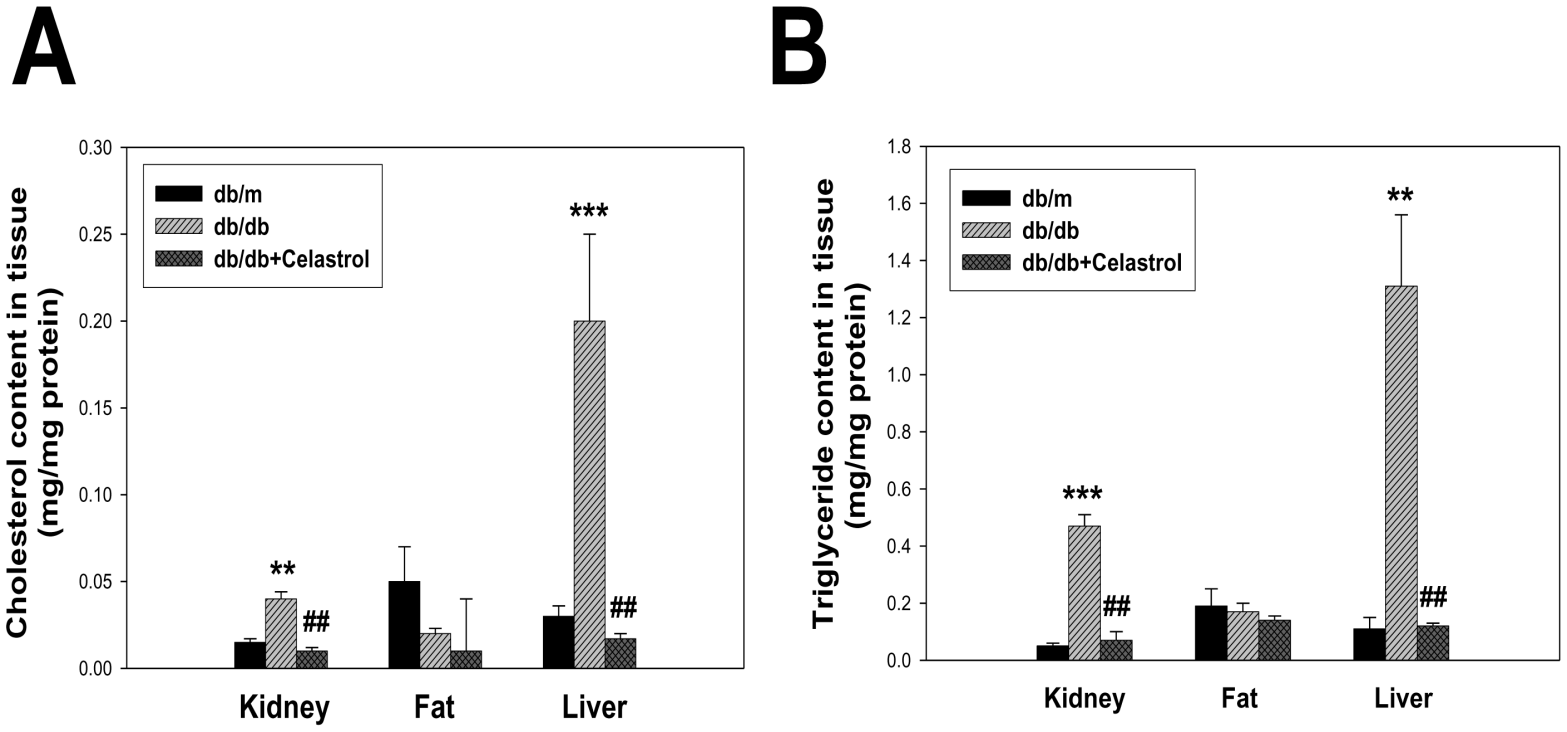
Effects of celastrol on tissue lipid accumulation in experimental animals. (A) Cholesterol contents in renal, adipose, and hepatic tissues. (B) Triglyceride contents in renal, adipose, and hepatic tissues. Data are shown as the mean±SEM; **p<0.01, ***p<0.001 vs. *db/m* group; ##p<0.01 vs. control *db/db* group.

**Figure 8:**
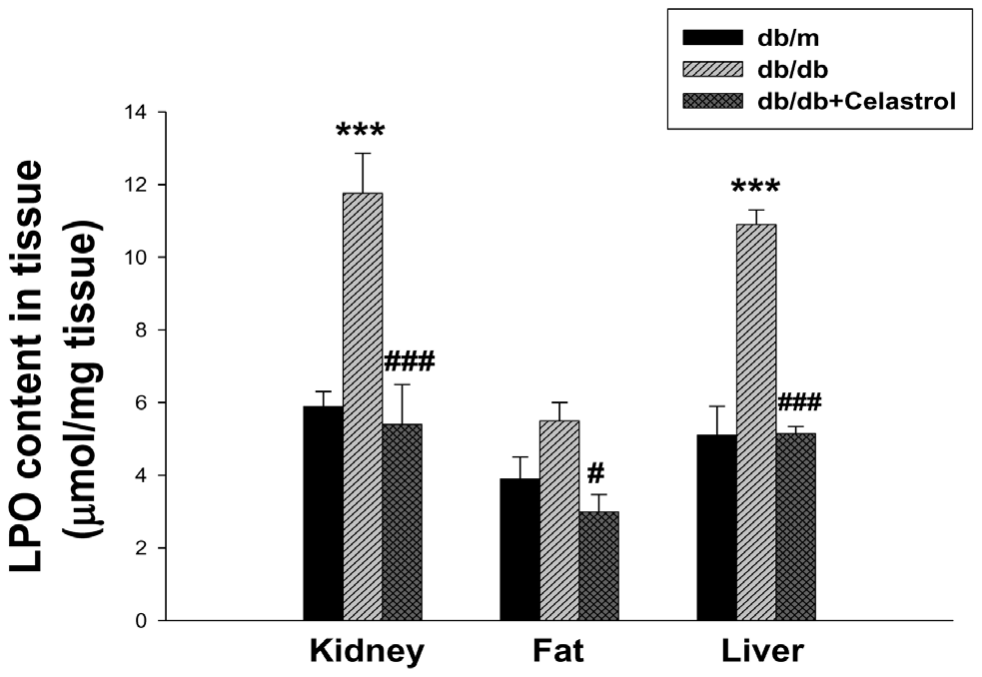
Effects of celastrol on lipid peroxidation in experimental animals. Data are shown as the mean±SEM; ***p<0.001 vs. *db/m* group; #p<0.05, ###p<0.001 vs. control *db/db* group.

Because celastrol administration improved microalbuminuria and insulin resistance and was related to improvements in metabolic dysfunction, we next investigated whether these improvements were related to a correction of lipotoxicity. As shown in Figure 7, cholesterol and triglyceride levels in renal and hepatic tissues were markedly increased in diabetic *db/db* mice than non-diabetic *db/m* mice, and significantly decreased by celastrol treatment, although these parameters did not show any significant differences in adipose tissue. Since oxidative stress in the diabetic condition induces the peroxidation of lipids and leads to cellular dysfunction, we also evaluated changes in LPO in tissues. LPO levels in the kidney, liver, and adipose tissues were significantly decreased by celastrol treatment (Figure 8).

### Effects of celastrol on proinflammatory cytokine synthesis in cultured podocytes

**Figure 9:**
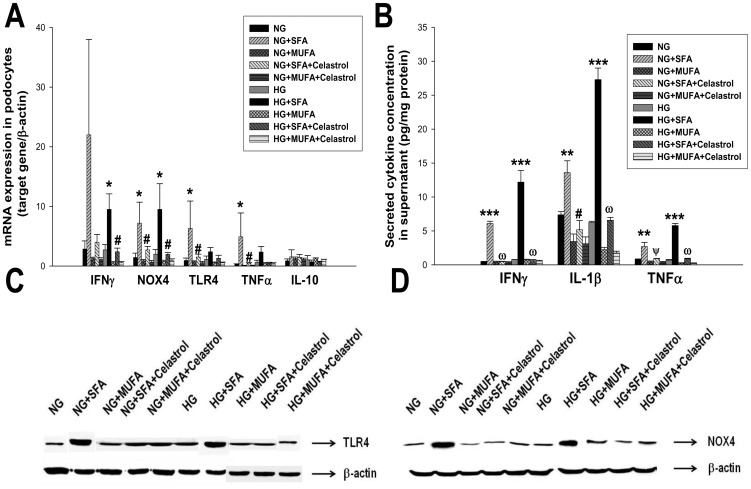
Effects of celastrol on inflammatory molecules in cultured podocytes. (A) mRNA expression in podocytes. (B) Secreted cytokine concentration in supernatant. Data are shown as the mean±SEM; (C) Representative western blot for TLR4 in podocytes. (D) Representative western blot for NOX4 in podocytes. SFA, saturated fatty acid; MUFA, monounsaturated fatty acids; *p<0.05, **p<0.01, ***p<0.001 vs. NG or HG; #p<0.05, Ψp<0.01, ω<0.0001 vs. SFA treated group.

Finally, we performed an *in vitro* experiment to further evaluate the direct effect of celastrol treatment in terms of anti-inflammatory effects in cultured podocytes. As shown in Figure 9A, stimulation with high glucose alone did not induce significant increases in the gene expression of inflammatory molecules including IFNγ, NOX4, TLR4, and TNFα, whereas stimulation with the palmitate (SFA) markedly up-regulated the expressions of these cytokine genes. Prior treatment with celastrol almost completely suppressed this SFA-induced cytokine molecule expression. However, oleic acid (MUFA) showed down-regulated tendency in inflammatory cytokine gene expression, although it did not reach statistical significance. However, there was no significant change in anti-inflammatory cytokines such as IL-10. In accordance with gene expression, inflammatory cytokine secretion was profoundly increased only after SFA stimulation and celastrol treatment completely abrogated fatty acid-induced inflammatory cytokine production (Figure 9B). In addition, NOX4 and TLR4 protein expression determined by western blot also showed similar changes (Figure 9C, 9D).

## Discussion

In the present study, we demonstrated that celastrol treatment significantly improved insulin resistance, glycemic control and metabolic parameters in *db/db* mice. Celastrol treatment also significantly mitigated lipid accumulation and oxidative stress in various organs including the liver and adipose tissue. In addition, celastrol treatment exhibited renal protective effects through improvement in renal lipid accumulation, oxidative stress and proinflammatory cytokine activity in the kidney. We also provided evidence that celastrol treatment abolished free fatty acid-induced proinflammatory cytokine synthesis in cultured podocytes.

The NF-κB signaling pathway is a central axis in tissue inflammation because NF-κB controls the expression of genes including inflammatory cytokines, chemokines, and adhesion molecules, all of which play pivotal roles in controlling inflammation [9]. Due to this attractive characteristic, targeted therapy for the NF-κB pathway has been of interest in the treatment of many inflammatory diseases. Although a great deal of evidence suggests the indirect renoprotective effect of various drugs and drug targets such as RAS blockade, ROS inhibition, alpha-tocopherol, and PPARα and PPARγ agonists secondary to inhibition of the NF-κB pathway [11], [13], [23], [24], [25], [26], [27], there is little evidence suggesting that direct inhibition of the NF-κB pathway has a beneficial effect in diabetic nephropathy. Furthermore, increasing data suggest an important role of the NF-κB pathway in the development of insulin resistance associated with adipose tissue inflammation [9], [28] and there is also some evidence regarding the effect of NF-κB inhibition on insulin resistance.

In the present study, we investigated the effects of celastrol, an NF-κB inhibitor, on insulin resistance and diabetic nephropathy in type 2 diabetic mice that are a well-known animal model of insulin resistance and nephropathy. We found that celastrol treatment markedly improved glycemic control and reduced HbA1C levels and HOMA-IR index scores in these overtly diabetic insulin resistant mice. Improvements in insulin resistance were further confirmed by insulin tolerance test. Celastrol-treated animals exhibited much better lipid profiles, including decreases in total cholesterol and triglyceride levels associated with improvement in hepatic steatosis.

In agreement with these results, recent studies have explored potential mechanisms of the NF-κB pathway in the pathogenesis of insulin resistance. Women with polycystic ovarian syndrome demonstrate insulin resistance and increases in NF-κB activation in mononuclear cells [29]. NF-κB DNA-binding activity and IkBα protein levels were significantly more elevated in PBMCs from type 2 diabetic patients than in non-diabetic controls [30]. In addition, NF-κB binding was positively associated with both body mass index and the homeostasis model assessment of insulin resistance in type 2 diabetic patients [31]. Genetic polymorphism in the 3' region of the IkBα gene have been associated with insulin resistance in Hispanic Americans in the Insulin Resistance Atherosclerosis (IRAS) Family Study [32]. Taken together, this data suggests a possible link between NF-κB activation and insulin resistance.

Celastrol treatment significantly decreased body weight due to decreases in food consumption. These results contrast with those of a previous study in animal models of infection-associated anorexia and NF-κB transgenic mice [33], [34]. Infection-related anorexia and weight loss are mediated via NF-κB activation in hypothalamic pro-opiomelanocortin (POMC) neurons. In addition, hypothalamic NF-κB was activated by leptin, an important anorexigenic hormone, and mediates leptin-stimulated POMC transcription [33]. Tang et al. reported that energy expenditure was elevated without a change in food intake in NF-κB transgenic mice [34]. Although it is not clear why the celastrol group showed lower body weight and decreased food intake, recent studies suggest that inhibition of NF-κB by celastrol in the hypothalamus may decrease levels of neuropeptide Y (NPY), which is an orexigenic hormone, resulting in decreased food intake and body weight. NPY is the most abundant neuropeptide within the central nervous system and its effects are mediated through the Y1 receptor, where it participates as an orexigenic hormone in the control of food intake [35]. Musso et al. reported that the Y1 receptor for NPY may represent one of the κB site-containing genes that is modulated by κB-related factors and acts as an enhancer element, inferring its potential role in regulating the expression of the Y1 receptor gene [36]. Furthermore, Zhang et al. also demonstrated that IKKβ/NF-κB remains inactive in hypothalamic neurons; however, overnutrition atypically activates hypothalamic IKKβ/NF-κB through elevated endoplasmic reticulum stress in the hypothalamus and activates hypothalamic AGRP neurons, leading to increased production of orexigenic hormones [37]. However, the potential adverse events such as decrease in food intake and body weight in healthy subjects should be considered before clinical application of celastrol as a new therapeutic strategy for treatment of diabetic nephropathy.

Obese adipose tissue has been proposed to be a pivotal organ in insulin resistance, whereby inflammatory processes such as infiltration of macrophages and increased cytokine synthesis occur, inducing systemic insulin resistance [17]. However, the physiologic action of NF-κB inhibition in adipose tissue remains uncertain.

In the present study, celastrol treatment significantly decreased epididymal fat masses and induced phenotypic changes to small differentiated adipocytes, which is a more insulin sensitive phenotype [38]. In addition, celastrol also markedly increased the adiponectin gene expression that is associated with increased plasma levels of adiponectin, all of which are related with improved insulin resistance. These results agree with those of a previous study that demonstrated that NF-κB levels were higher in mice treated with a high fat diet, and that inhibition of NF-κB restored the lipotoxicity of adipose tissue and decreased fat mass [39]. Furthermore, celastrol treatment significantly decreased the levels of SFAs, and increased the levels of MUFAs in hepatic and adipose tissue, that are more anti-inflammatory fatty acid composition. We also observed that adipose tissue lipid peroxidation was markedly decreased in the celastrol-treated group. Taken together, these results imply that NF-κB inhibition decreased inflammation and oxidative stress in adipose tissue and led to improved insulin resistance.

The most important finding of this study is that celastrol treatment decreased the urinary excretion of albumin and plasma creatinine levels. Additionally, celastrol decreased mesangial expansion in renal tissues, accompanied by suppression of the synthesis of profibrotic and proinflammatory molecules. We observed that NF-κB activation was increased in diabetic *db/db* mice compared with that in control db/m mice. Celastrol treatment decreased renal NF-κB activation in db/db mice. In addition, celastrol treatment markedly inhibited NOX4 expression, a marker of oxidative stress in the kidney, and decreased the urinary excretions of 8-isoprostane, a marker of oxidative stress, and inflammatory cytokines including TNFα and IL-2. Taken together, these results suggest that celastrol treatment inhibited oxidative stress and inflammatory processes in the kidney. These results agree with those of a previous report that concluded celastrol treatment attenuates hypertension-induced inflammation and oxidative stress in fructose-induced hypertensive rats [40].

Another interesting finding of this study is the improvement in renal lipid metabolism by celastrol treatment. In diabetic mice, mRNA expression levels of enzymes involved in cholesterol synthesis, such as HMG-CoA reductase and SREBP1c, were significantly increased in diabetic db/db mice compared with those in control *db/m* mice (data not shown). Celastrol treatment markedly decreased renal accumulation of cholesterol and triglyceride content in renal cortical tissues associated with the suppression of renal LPO content. This finding agrees with previous reports that suggest the role of abnormal renal lipid metabolism in the pathogenesis of diabetic nephropathy [41], [42]. These results suggest that NF-κB inhibition in part improves renal function via improvements in renal lipid metabolic abnormalities.

In terms of decreased tissue levels of LPO, it may be possible that decreased tissue accumulation of lipid such as cholesterol and triglyceride may decrease the LPO contents in various organs instead of direct celastrol effects. To elucidate this point, we performed another in vivo experiment using *db/m mice*. As shown in Table S2, 2weeks treatment of celastrol significantly decreased tissue LPO contents in various organs without significant difference in tissue lipid accumulation. These results suggest that decreased tissue LPO contents are due to the direct effects of celastrol instead of lipotoxicity.

In the present study, we performed *in vitro* experiments to further elucidate the role of celastrol on diabetic nephropathy, and found that high glucose alone did not induce significant upregulation of inflammatory cytokines and oxidative stress markers, whereas free fatty acid treatment profoundly upregulated these molecules. We observed that celastrol abolished this free fatty acid-induced inflammatory and oxidative stress molecule synthesis. Considering the important role of free fatty acids on diabetic glomerular injury [43], these results suggest that decreased renal inflammation and oxidative stress by NF-κB inhibition resulted in improvement in renal lipid metabolism, leading to a partial contribution to the renoprotective effects of celastrol.

In conclusion, the NF-κB inhibitor celastrol provided a protective effect against target organ damage in type 2 diabetic mice through improved metabolic alterations as well as inhibition of profibrotic and proinflammatory processes in the target organs. These findings suggest that the NF-κB pathway may be a useful new therapeutic target in the treatment of type 2 diabetes mellitus and diabetic nephropathy.

## Supporting Information

Figure S1 **Effects of celastrol on histologic changes in adipose and hepatic tissues.** (A, B, C) adipose tissue, (D, E, F) hepatic tissue, (A, D, *db/m*; B, E, *db/db*; C, F, *db/db*+celastrol. Original magnification X200.
(TIF)

Table S1 **Primer sequences for real-time quantitative PCR.**
(DOCX)

Table S2 **Physical and biochemical parameters of experimental animals.**
(DOCX)

Table S3 **Fatty acid composition in various organs in experimental animals.**
(DOCX)
