# Supplementary figures and images for: Celastrol, an NF-κB Inhibitor, Improves Insulin Resistance and Attenuates Renal Injury in db/db Mice
Source: PLoS One. 2013 Apr 26;8(4):e62068. doi: 10.1371/journal.pone.0062068 (PMC3637455; doi:10.1371/journal.pone.0062068)

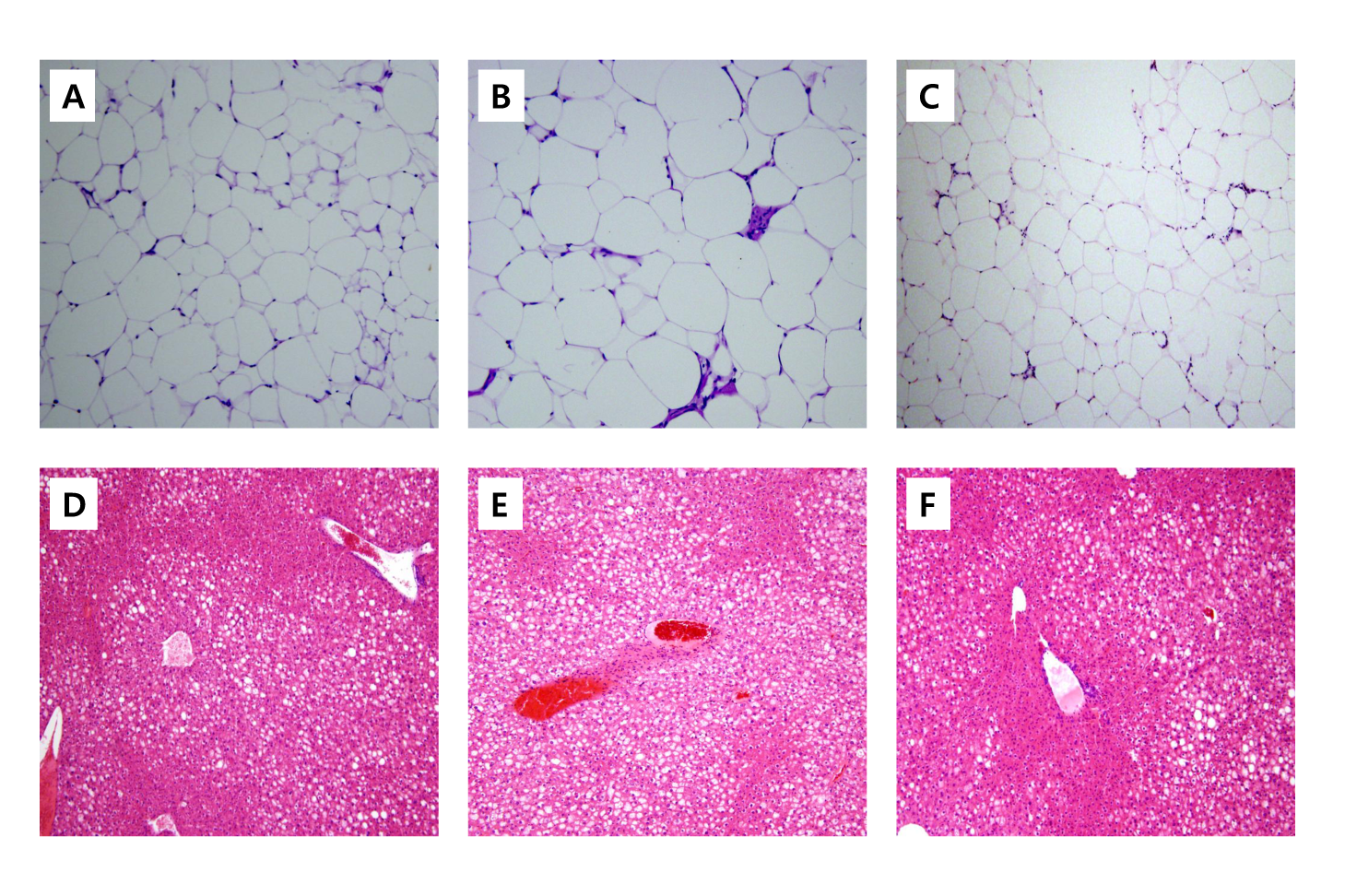

Supplement: Figure S1 — Effects of celastrol on histologic changes in adipose and hepatic tissues. (A, B, C) adipose tissue, (D, E, F) hepatic tissue, (A, D, db/m; B, E, db/db; C, F, db/db+celastrol. Original magnification X200. (TIF) [file pone.0062068.s001.tif]
