# Supplementary material for: Celastrol, an NF-κB Inhibitor, Improves Insulin Resistance and Attenuates Renal Injury in db/db Mice
Source: PLoS One. 2013 Apr 26;8(4):e62068. doi: 10.1371/journal.pone.0062068 (PMC3637455; doi:10.1371/journal.pone.0062068)
Supplement: Table S1 — Primer sequences for real-time quantitative PCR. (DOCX) [file pone.0062068.s002.docx]

### **Table S1. Primer sequences for real-time quantitative PCR**

| Target gene | Primer sequence (5’ to 3’) | Amplicon length(bp) |
| --- | --- | --- |
| IFNγ , forward | GCGTCATTGAATCACACCTG | 129 |
| IFNγ , reverse | TGAGCTCATTGAATGCTTGG |  |
| TNFα, forward | CCGATGGGTTGTACCTTGTC | 131 |
| TNFα, reverse | GGCAGAGAGGAGGTTGACTTT |  |
| IL-10, forward | ATCGATTTCTCCCCTGTGAA | 93 |
| IL-10, reverse | TGGCCTTGTAGACACCTTGG |  |
| NOX-4, forward | TTGGTGAATGCCCTCAACTT | 88 |
| NOX-4, reverse | TTCTGGGATCCTCATTCTGG |  |
| TLR4, forward | GGGAACAAACAGCCTGAGAC | 75 |
| TLR4, reverse | AGACCCATGAAATTGGCAT |  |
| β -actin, forward | GGACTCCTATGTGGGTGACG | 118 |
| β -actin, reverse | CTTCTCCATGTCGTCCCAGT |  |

IFNγ, interferon γ; TNFα, tumor necrosis factor α; IL-10, interleukin-10; NOX-4, NADPH oxidase-4; TLR4, toll-like receptor 4. In this experiment, each sample was run in triplicate, and the corresponding non-reverse transcribed mRNA samples were used as negative controls. The mRNA level of each sample was normalized to that of β-actin mRNA.
