# Supplementary material for: Celastrol, an NF-κB Inhibitor, Improves Insulin Resistance and Attenuates Renal Injury in db/db Mice
Source: PLoS One. 2013 Apr 26;8(4):e62068. doi: 10.1371/journal.pone.0062068 (PMC3637455; doi:10.1371/journal.pone.0062068)
Supplement: Table S2 — Physical and biochemical parameters of experimental animals. (DOCX) [file pone.0062068.s003.docx]

**Table S2. Physical and biochemical parameters of experimental animals**

| Parameters | week | *db/m* control | *db/m* control+celastrol |
| --- | --- | --- | --- |
| Body weight (g) | 0 | 21.88±0.55 | 22.18±0.26 |
|  | 2 | 23.11±0.36 | 19.09±0.31^**^ |
| Daily Food intake (g) | 0 | 2.62±0.09 | 2.72±0.04 |
|  | 2 | 3.25±0.08 | 1.92±0.06^**^ |
| Daily water intake (g) | 0 | 3.83±0.19 | 3.76±0.09 |
|  | 2 | 4.86±0.09 | 4.36±0.17 |
| Fasting plasma glucose (mmol/l) | 0 | 7.37±0.3 | 7.29±0.2 |
|  | 2 | 8.2±0.5 | 7.4±0.5^*^ |
| Urine volume (ml/day) | 0 | 0.34±0.09 | 0.43±0.15 |
|  | 2 | 0.49±0.12 | 0.43±0.15 |
| Kidney/100g BW | 2 | 1.20±0.28 | 1.32±0.02 |
| Heart/100g BW | 2 | 0.45±0.01 | 0.51±0.02 |
| Fat/100g BW | 2 | 1.51±0.11 | 0.67±0.12^**^ |
| Liver/100g BW | 2 | 4.90±0.09 | 5.15±0.29 |
| Plasma creatinine (μmol/l) | 2 | 26.1±2.0 | 25.4±2.1 |
| Plasma cholesterol (mg/dL) | 2 | 72±14 | 66±22 |
| Plasma triglyceride (mg/dL) | 2 | 46±12 | 42±19 |
| UAE (μg/mgCr/day) | 2 | 17.02±6.23 | 11.15±2.07 |
| Kidney Chol content (mg/mg protein) | 2 | 0.014±0.01 | 0.016±0.01 |
| Liver Chol content (mg/mg protein) | 2 | 0.011±0.01 | 0.012±0.01 |
| Fat Chol content (mg/mg protein) | 2 | 0.025±0.01 | 0.021±0.01 |
| Kidney TG content (mg/mg protein) | 2 | 0.057±0.03 | 0.051±0.03 |
| Liver TG content (mg/mg protein) | 2 | 0.144±0.04 | 0.165±0.08 |
| Fat TG content (mg/mg protein) | 2 | 0.241±0.07 | 0.23±0.07 |
| Kidney LPO content (μmol/mg tissue) | 2 | 5.39±1.12 | 3.25±1.11^*^ |
| Liver LPO content (μmol/mg tissue) | 2 | 8.39±1.21 | 6.55±1.88^*^ |
| Fat LPO content (μmol/mg tissue) | 2 | 8.31±1.73 | 4.49±0.34^*^ |

Values are expressed as means ± SEM. Statistical analysis was performed between groups at the same time periods; UAE, urinary albumin excretion; Chol, cholesterol; TG, triglyceride; LPO, lipid hydroperoxides; *P<0.05; **P<0.01 vs. *db/m* control.
