# Supplementary material for: Celastrol, an NF-κB Inhibitor, Improves Insulin Resistance and Attenuates Renal Injury in db/db Mice
Source: PLoS One. 2013 Apr 26;8(4):e62068. doi: 10.1371/journal.pone.0062068 (PMC3637455; doi:10.1371/journal.pone.0062068)
Supplement: Table S3 — Fatty acid composition in various organs in experimental animals. (DOCX) [file pone.0062068.s004.docx]

**Table S3**. **Fatty acid composition in various organs in experimental animals.**

| Composition of fatty acids (mg/kg tissue) | | *db/m* control  (% of total FAs) | | *db/db* + vehicle  (% of total FAs) | | *db/db* + celastrol  (% of total FAs) | |
| --- | --- | --- | --- | --- | --- | --- | --- |
|  | Name of fatty acids | Fat | Liver | Fat | Liver | Fat | Liver |
| SFA | Myristic Acid (C14:0) | 1.75±0.11 | N.D. | 1.95±0.21 | N.D. | 1.51±1.2 | N.D. |
|  | Pentadecanoic Acid (15:0) | 0.51±0.12 | N.D. | 1.32±0.14 | N.D. | N.D. | N.D. |
|  | Palmitic Acid (C16:0) | 27.77±2.12 | 10.95±4.5 | 39.75±5.9 | 32.25±9.7 | 19.93±3.4 | 15.51±2.3 |
|  | Stearic Acid (C18:0) | 2.82±1.23 | 0.4±0.23 | 7.05±2.34 | 6.5±1.5 | 3.43±1.3 | 4.25±1.6 |
|  | Heptadecanoic Acid (C17:0) | 1.2±0.11 | N.D. | N.D. | N.D. | 0.22±0.11 | N.D. |
|  | Behenic Acid (C22:0) | 1.2±0.15 | N.D. | 0.23±0.21 | N.D. | N.D. | N.D. |
|  | **% of SFA in total FAs** | **35.25±1.7** | **11.35±3.2** | **50.3±3.8^**^** | **38.75±6.7^***^** | **25.09±2.2^*,^**^¶^ | **19.76±2.1**^¶^ |
| MUFA | Palmitoleic Acid (C16:1) | 4.76±0.23 | N.D. | 5.39±2.31 | 3.44±1.2 | 13.89±3.2 | 7.19±3.2 |
|  | Cis-10-Heptadecenoic Acid (C17:1) | 1.16±0.12 | N.D. | 1.9±1.1 | N.D. | 0.52±0.2 | N.D. |
|  | Elaidic Acid (C18:1n-9,trans) | N.D. | N.D. | N.D. | N.D. | 0.22±0.1 | N.D. |
|  | Oleic Acid (C18:1n-9,cis) | 16.09±1.16 | N.D. | 16.22±3.4 | 25.51±3.4 | 30.32±5.6 | 46.9±6.9 |
|  | Cis-11-Eicosenoic Acid (C20:1) | N.D. | N.D. | N.D. | N.D. | 0.32±0.1 | N.D. |
|  | **% of MUFA in total FAs** | **22.01±1.31** | **N.D.** | **23.51±2.5** | **28.95±2.1** | **45.27±2.2^***,^**^¶^ | **54.09±5.7**^¶^ |
| n-3 PUFA | Linolenic Acid (C18:3n-3) | N.D. | N.D. | 1.23±0.21 | 0.16±0.12 | 0.85±0.3 | N.D. |
|  | Tricosanoic Acid (C23:0) | 0.1±0.01 | 8.68±3.14 | N.D. | 2.48±1.1 | N.D. | 1.78±0.2 |
|  | Cis-5,8,11,14,17-Eicosapentaenoic Acid (C20:5n-3) | N.D. | N.D. | 0.16±0.04 | N.D. | N.D. | N.D. |
|  | Cis-4,7,10,13,16,19-Docosahexaenoic Acid (C22:6n-3) | N.D. | 7.59±2.67 | N.D. | 2.87±1.3 | N.D. | 0.56±0.12 |
|  | **% of n-3 PUFA in total FAs** | **0.1±0.01** | **16.27±3.1** | **1.39±0.12^**^** | **5.51±0.2^*^** | **0.85±0.3^*,^**^†^ | **2.34±0.15** |
| n-6 PUFA | Linoleic Acid (C18:2n-6,cis) | 40.49±5.67 | 72.3±22.1 | 24.55±4.9 | 26.79±3.4 | 28.79±3.3 | 23.81±5.45 |
|  | Cis-11,14-Eicosadienoic Acid (C20:2) | 1.02±0.12 | N.D. | N.D. | N.D. | N.D. | N.D. |
|  | Cis-8,11,14-Eicosatrienoic Acid (C20:3n-6) | 1.13±0.16 | N.D. | 0.25±0.11 | N.D. | N.D. | N.D. |
|  | **% of n-6 PUFA in total FAs** | **42.64±2.78** | **72.38±22.1** | **24.8±2.1^***^** | **26.79±3.4^***^** | **28.79±3.3^**^** | **23.81±5.4** |

Values are expressed as means ± SEM. Statistical analysis was performed among same organs; *P<0.05; **P<0.01; ***P<0.001 vs. *db/m* control; †P<0.05; ¶ P<0.001 vs. *db/db* + vehicle; MUFA, monounsaturated FAs; PUFA, polyunsaturated FAs; SFA, saturated FAs.
